# GAC-BiT-CNN: A Hybrid Generative Adversarial Capsule–Transformer–CNN Model with Fused Protein Language Model Embeddings for Methyltransferase Prediction

**DOI:** 10.3390/ijms27219967

**Published:** 2026-11-06

**Authors:** Muhammad Muazzam, Farman Ali, Raed Alsini, Naif Waheb Rajkhan, Omar Alghushairy, Tamim Alkhalifah

**Affiliations:** 1 Department of Computer Science, Bahria University, Islamabad 44000, Pakistan; muazzammuhammad043@gmail.com; 2 Department of Information Systems, Faculty of Computing and Information Technology, King Abdulaziz University, Jeddah 21589, Saudi Arabia; 3 Department of Computer Science, Faculty of Computing and Information Technology, King Abdulaziz University, Jeddah 21589, Saudi Arabia; 4 Department of Information Systems and Technology, College of Computer Science and Engineering, University of Jeddah, Jeddah 23890, Saudi Arabia; 5 Department of Information Technology, College of Computer, Qassim University, Buraydah 52571, Saudi Arabia

**Keywords:** methyltransferases, protein language models, ESM-2, ProtT5-XL, SHAP

## Abstract

Methyltransferases (MTs) are a functionally diverse class of enzymes that catalyse methyl group transfer from S-adenosyl-L-methionine (SAM) to DNA, RNA, and proteins, thereby regulating epigenetic programming, gene expression, and numerous disease processes. Dysregulation of these enzymes is implicated in a wide spectrum of human cancers and neurodegenerative disorders, making their accurate computational identification a priority in both basic research and drug discovery. We present GAC-BiT-CNN, a novel hybrid deep learning framework integrating Generative Adversarial Capsule Networks (GAC), Bidirectional Transformers (BiT), and Convolutional Neural Networks (CNN). The model leverages high-dimensional protein embeddings from four protein language models, including ProtGen, ESM-2, ProtT5-BFD, and ProtT5-XL, used individually, together with a fused feature set formed by concatenating the ProtT5-BFD and ProtT5-XL embeddings. All architectural components were optimised via systematic grid search hyperparameter tuning, and SHapley Additive exPlanations (SHAP)-based analysis was applied to the fused embeddings to identify the most discriminative feature dimensions. GAC-BiT-CNN with the fused feature set achieved 98.34% accuracy on the training set and 95.50% accuracy on the held-out test set, with an area under the receiver operating characteristic curve (AUC) of 98.70% and a Matthews correlation coefficient (MCC) of 0.901, surpassing all baseline models by a substantial margin. GAC-BiT-CNN provides a scalable, alignment-free framework with representation-level SHAP attribution for high-throughput methyltransferase identification, with direct applications in epigenomics, drug discovery, and cancer biology.

## 1. Introduction

**Figure 1:**
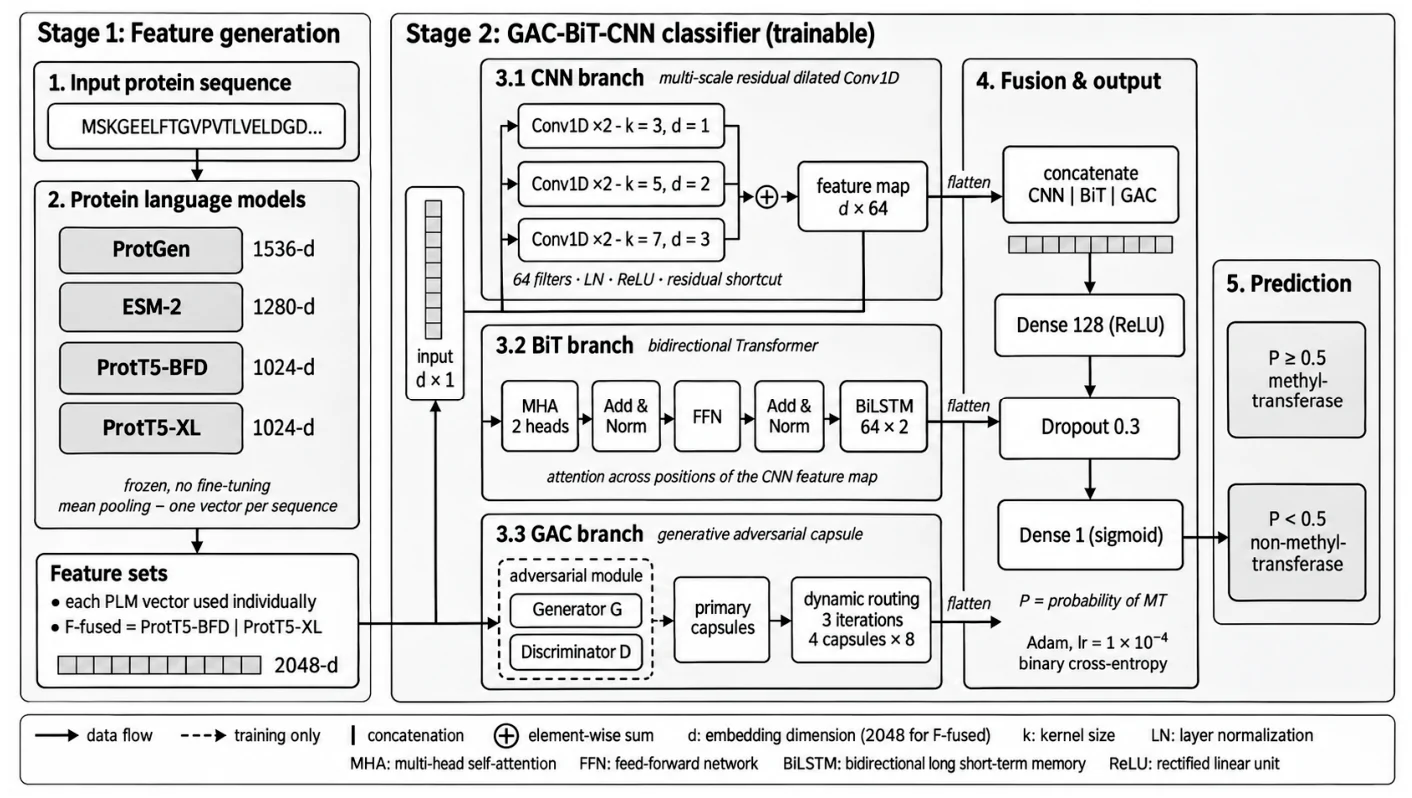
Overall architecture of the proposed GAC-BiT-CNN model for methyltransferase prediction. The pipeline consists of: (1) protein sequence input; (2) embedding generation via four protein language models; (3) three processing branches, including CNN for local pattern extraction, and BiT for long-range dependencies and GAC for adversarial capsule-based hierarchical features, both operating on the CNN feature map; (4) feature fusion by concatenation; and (5) binary classification output. Steps (1)–(2) form Stage 1 (feature generation and fusion with frozen PLMs) and steps (3)–(5) form Stage 2 (the trainable GAC-BiT-CNN classifier). CD-HIT (Cluster Database at High Identity with Tolerance), a program that groups sequences sharing more than a chosen level of sequence identity and keeps one representative of each group, was used only to remove redundant sequences when the benchmark dataset was constructed and is not part of the prediction framework.

Methyltransferases (MTs) are a ubiquitous class of enzymes that catalyse the transfer of methyl groups from the universal methyl donor S-adenosyl-L-methionine (SAM) to diverse biological substrates, including DNA, RNA, and proteins. This enzymatic reaction underpins a wide range of cellular processes, including chromatin remodelling, transcriptional regulation, RNA processing, protein stability, and small-molecule biosynthesis [1,2]. Classified into DNA methyltransferases (DNMTs), RNA methyltransferases (RNMTs), and protein methyltransferases (PMTs), the latter further subdivided into protein arginine methyltransferases (PRMTs) and protein lysine methyltransferases (PKMTs), these enzymes constitute a functionally diverse superfamily whose activity is tightly coupled to development, differentiation, and adaptive cellular responses [3,4]. Beyond their fundamental physiological roles, methyltransferases have substantial clinical relevance. Dysregulation and aberrant expression of PRMTs and PKMTs are causally linked to the initiation and progression of numerous human malignancies, including breast, lung, colorectal, and haematological cancers [5,6]. Several small-molecule PMT inhibitors are currently undergoing clinical trials as anti-cancer agents [7,8]. Beyond oncology, aberrant methylation has been implicated in neurodegenerative conditions such as Alzheimer’s and Parkinson’s disease, as well as in cardiovascular and pulmonary disorders [9,10]. Collectively, this broad disease relevance has elevated methyltransferases to high-priority therapeutic targets across multiple clinical contexts. At the same time, the wide distribution of methyltransferases poses a challenge for drug development. Because most methyltransferases share a conserved SAM-binding site, an inhibitor designed against one enzyme may also inhibit non-target methyltransferases and cause side effects. Selectivity is therefore a central requirement, and current inhibitors achieve it mainly by binding the less conserved substrate-binding site or allosteric sites, with selectivity assessed against panels of related methyltransferases [7,8,9]. The method presented here does not design inhibitors; its role is limited to identifying methyltransferases among uncharacterised sequences. Conventional experimental approaches for identifying and characterising methyltransferases, including mass spectrometry, X-ray crystallography, and nuclear magnetic resonance spectroscopy, remain the gold standard for biochemical validation. However, these techniques are inherently low-throughput, resource-intensive, and impractical for the large-scale annotation of the rapidly expanding sequence databases [9]. Consequently, the vast majority of putative methyltransferase sequences deposited in databases such as the National Centre for Biotechnology Information (NCBI) and the UniProt Knowledgebase (UniProtKB) remain without in vitro characterisation [11]. This annotation bottleneck underscores the urgent need for accurate, scalable, and alignment-independent computational tools for methyltransferase identification. Computational prediction of methyltransferases has evolved considerably in recent years. Early methods relied on sequence profile techniques, hidden Markov models (HMMs), and motif searches, which perform poorly on divergent enzyme families with low inter-subfamily sequence similarity [12]. The latest and most prominent example is PMTPred by Yadav et al. [13], which employs support vector machines (SVMs) trained on the composition of k-spaced amino acid pairs (CKSAAP) features, achieving an accuracy of 87.94%. Deep learning (DL) methods such as DEEP-EP [14], UniproLcad [15], and CNN-BiEP [16] have been applied to related protein-function prediction tasks. Nevertheless, the literature lacks a dedicated DL architecture for methyltransferase prediction that exploits recently developed protein language model (PLM) embeddings. To address this gap, we introduce GAC-BiT-CNN, a hybrid deep learning framework that integrates: (i) Generative Adversarial Capsule Networks (GAC) for learning spatial and hierarchical features via adversarial training; (ii) Bidirectional Transformers (BiT) for modelling long-range dependencies across the learned feature representation; and (iii) Convolutional Neural Networks (CNN) for learning local patterns from the fused embedding. The model is trained on four PLM embeddings, i.e., ProtGen, ESM-2 [17], ProtT5-BFD, and ProtT5-XL [18] embeddings, used individually and, for ProtT5-BFD and ProtT5-XL, combined into a fused feature set, to enable end-to-end, alignment-free feature extraction directly from raw amino acid sequences. The framework has two stages: in Stage 1, the PLMs are used as frozen feature extractors, and the mean-pooled ProtT5-BFD and ProtT5-XL embeddings are concatenated into one fused vector; in Stage 2, this vector is classified by the trainable GAC-BiT-CNN network. The task addressed in this study is the binary classification of protein sequences as methyltransferases (MTs, acting on any substrate) or non-methyltransferases; protein methyltransferases (PMTs) are one clinically important subgroup within the positive class [19,20]. The intended use of GAC-BiT-CNN should be stated precisely. The method is not designed to identify the sequences of human methyltransferases that are already established drug targets, as these are characterised experimentally. Instead, it addresses the annotation of sequences whose function is unknown. First, genome and metagenome sequencing produces far more protein sequences than can be characterised experimentally, including sequences from microorganisms and pathogens that have no close, experimentally characterised homologue. Second, homology-based annotation transfer, the conventional first step, becomes unreliable when sequence identity to characterised proteins is low, because enzyme function is less conserved than overall sequence similarity [21]; in such cases a homology search may return no hit, or a hit whose own function was inferred computationally, which can propagate annotation errors [11]. Third, even the human methyltransferasome contains predicted methyltransferases whose activity has not yet been confirmed [12,20]. GAC-BiT-CNN is therefore intended as a fast, alignment-free screen that complements, rather than replaces, homology search and experimental assays: it can prioritise candidate methyltransferases among uncharacterised sequences for experimental validation, provide a second opinion when homology search is inconclusive, and flag sequences whose existing annotation may be incorrect. The schematic view of the proposed study is listed in Figure 1.

## 2. Results

### 2.1. Performance of DL Models on the Training Dataset

In this study, we investigate the discriminative capacity of ProtGen, ESM-2, ProtT5-BFD, ProtT5-XL, and a feature-fused (F-fused) combination using a gated recurrent unit (GRU) network [22], a bidirectional long short-term memory (BiLSTM) network, a recurrent neural network (RNN), and the proposed GAC-BiT-CNN. Each classifier is evaluated under a five-fold cross-validation protocol, and performance is assessed using seven standard evaluation metrics: Accuracy (Acc), Sensitivity (Sn), Specificity (Sp), Precision (Pre), F1-score, Area Under the ROC Curve (AUC), and Matthews Correlation Coefficient (MCC). The consolidated results are presented in Table 1.

Using ProtGen features, GAC-BiT-CNN achieved the highest accuracy (96.42%), AUC (99.40%), and MCC (0.929) among all classifiers, substantially outperforming RNN (92.21%), BiLSTM (89.83%), and GRU (88.14%). RNN demonstrated competitive performance in this feature setting, surpassing both BiLSTM and GRU with an accuracy of 92.21% and MCC of 0.844, suggesting that even simple recurrent architectures can exploit ProtGen embeddings effectively. BiLSTM achieved a balanced performance with sensitivity of 89.91% and specificity of 89.75%, while GRU recorded the lowest accuracy (88.14%) and MCC (0.764) among all ProtGen-based configurations.

With ESM-2 features, a consistent performance drop was observed across all classifiers relative to other feature representations, indicating limited compatibility between ESM-2 embeddings and recurrent sequential architectures in this classification task. GRU recorded the weakest ESM-2 performance with an accuracy of 82.28% and MCC of 0.658, followed by BiLSTM (82.21%, MCC = 0.644), and RNN (85.93%, MCC = 0.718). In contrast, GAC-BiT-CNN achieved a remarkable accuracy of 97.84%, AUC of 99.76%, and MCC of 0.957 with ESM-2, demonstrating that its hybrid convolutional-attention architecture is uniquely capable of extracting discriminative patterns from ESM-2 representations that recurrent architectures fail to utilise effectively.

For the ProtT5-BFD descriptor, performance improved substantially across all classifiers relative to ESM-2. RNN achieved an accuracy of 92.21% and AUC of 97.70% with ProtT5-BFD, identical to its ProtGen performance, indicating that these two feature types are equally informative for recurrent models. BiLSTM recorded a moderate accuracy of 85.20% and MCC of 0.704, while GRU achieved its best performance among all features under ProtT5-BFD and ProtT5-XL (Acc = 88.93%, MCC = 0.779). GAC-BiT-CNN continued to demonstrate superior discriminative capability with an accuracy of 97.99%, AUC of 99.71%, and MCC of 0.960, further confirming the consistent advantage of the proposed architecture across diverse protein language model embeddings.

The ProtT5-XL descriptor yielded results nearly identical to ProtT5-BFD across all classifiers, reflecting the highly similar representational properties of these two ProtT5-derived embeddings. GRU achieved an accuracy of 88.93% and MCC of 0.779, matching its ProtT5-BFD performance, while RNN recorded 88.10% accuracy and MCC of 0.762, slightly below its ProtT5-BFD results. BiLSTM showed a marginal improvement over its ProtT5-BFD performance (85.26% vs. 85.20%), while GAC-BiT-CNN achieved its second-highest single-feature performance with an accuracy of 98.03%, AUC of 99.72%, and MCC of 0.961. The near-perfect concordance between ProtT5-BFD and ProtT5-XL results across all four classifiers confirms that these embeddings encode largely equivalent biological information under the evaluated architectures.

The F-fused feature set, constructed by combining multiple protein feature representations, delivered the strongest overall performance across the entire table, but exclusively in the context of GAC-BiT-CNN. GAC-BiT-CNN with F-fused features achieved the highest accuracy (98.34%), sensitivity (98.39%), specificity (98.29%), precision (98.31%), F1-score (98.34%), AUC (99.78%), and MCC (0.967) of any configuration evaluated in Table 1, confirming that multi-branch convolutional architectures are uniquely equipped to exploit complementary information encoded across heterogeneous feature spaces. By contrast, F-fused features offered little or no consistent benefit for GRU (Acc = 87.00%, MCC = 0.741), BiLSTM (Acc = 83.73%, MCC = 0.675), or RNN (Acc = 88.95%, MCC = 0.779) relative to their best individual feature configurations, suggesting that recurrent architectures lack the parallel multi-scale processing capacity necessary to effectively integrate fused feature representations.

**Figure 2:**
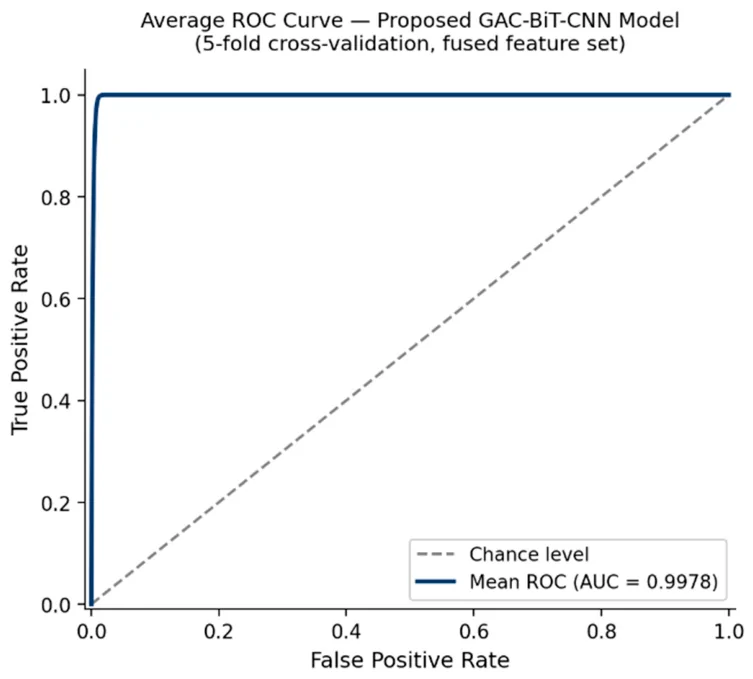
Average (mean) ROC curve of the proposed GAC-BiT-CNN model with the fused feature set on the training dataset.

To further assess the probabilistic discrimination capacity of all classifiers, Receiver Operating Characteristic (ROC) curves were generated in Figure 2 for each model under the F-fused feature setting, as it represents the most comprehensive and informative feature configuration. The ROC curves illustrate the trade-off between the True Positive Rate (TPR) and False Positive Rate (FPR) across all classification thresholds. GAC-BiT-CNN achieved the highest AUC of 99.78%, closely followed by RNN (95.19%), GRU (93.95%), and BiLSTM (91.42%) under F-fused features. All models demonstrated a characteristic sharp rise toward the upper-left corner of the ROC space, reflecting high sensitivity with minimal false positive rate. However, the substantially larger AUC margin of GAC-BiT-CNN over the three recurrent baselines underscores its superior ability to assign well-calibrated class probabilities, particularly in borderline sequence regions where recurrent models exhibit greater uncertainty.

Across all feature representations and evaluation metrics, GAC-BiT-CNN consistently and substantially outperformed GRU, BiLSTM, and RNN, with accuracy improvements ranging from approximately 6 to 16 percentage points and MCC improvements of 0.08 to 0.32 relative to the best recurrent baseline in each feature setting. These results collectively establish GAC-BiT-CNN as the most effective DL architecture for methyltransferase classification in this study, attributable to its integration of multi-scale convolutional feature extraction, attention-based contextual weighting, and the capacity to leverage complementary information across diverse protein language model embeddings.

### 2.2. SHAP-Based Interpretation of GAC-BiT-CNN on the Fused Feature Set

**Figure 3:**
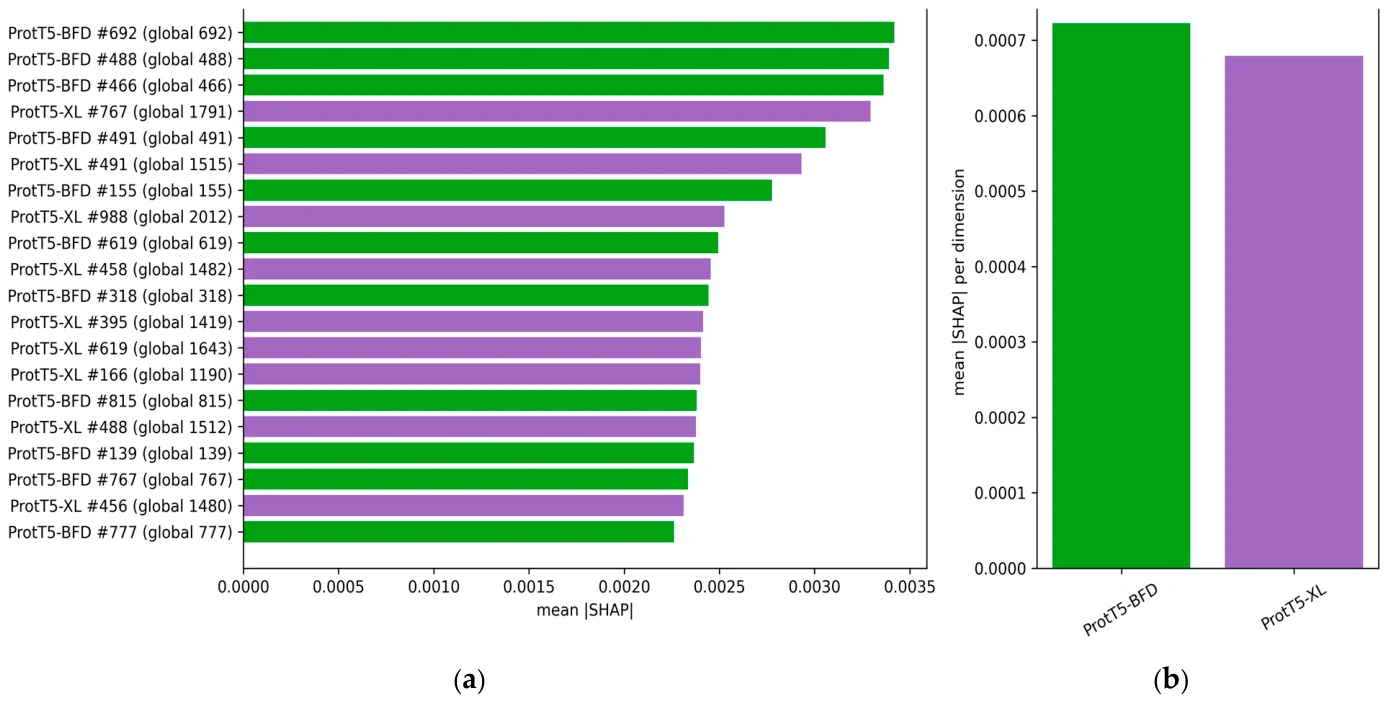
Mean absolute SHAP values for the top 20 most influential latent dimensions in the fused feature set (ProtT5-BFD∥ProtT5-XL, global indices 0–2047), computed with GradientExplainer on 300 test sequences. (**a**) Top 20 dimensions, labelled with the PLM of origin, local index and global index; (**b**) mean |SHAP| per dimension for each PLM block.

SHapley Additive exPlanations (SHAP) were applied [23] using the SHAP Python library (https://github.com/shap/shap, accessed on 4 October 2026) to provide model transparency and to identify the most influential features driving classifier performance across the fused feature set (F-fused = [ProtT5-BFD∥ProtT5-XL]). SHAP assigns each feature a contribution score grounded in cooperative game theory, quantifying the marginal impact of each latent dimension on the model output, thereby enabling both global model-level and local instance-level interpretability. The fused feature space was partitioned into two contiguous subspaces: ProtT5-BFD occupying dimensions 0–1023 and ProtT5-XL occupying dimensions 1024–2047. SHAP values were computed with the gradient explainer on the trained fused-feature model, using 100 training sequences as background and 300 test sequences for explanation. As shown in Figure 3, each bar is colour-coded according to its source descriptor, i.e., green for ProtT5-BFD and purple for ProtT5-XL, enabling direct visual attribution of each influential dimension to its originating protein language model. SHAP therefore explains which PLM-derived latent dimensions the classifier relies on; because each dimension is an abstract feature that combines information from all residues through mean pooling, it does not explain which residues are important.

Of the 20 most influential dimensions (Figure 3a), 11 originate from ProtT5-BFD (local indices 692, 488, 466, 491, 155, 619, 318, 815, 139, 767 and 777) and nine from ProtT5-XL (local indices 767, 491, 988, 458, 395, 619, 166, 488 and 456, i.e., global indices 1791, 1515, 2012, 1482, 1419, 1643, 1190, 1512 and 1480). The three highest-ranked dimensions all belong to ProtT5-BFD (global indices 692, 488 and 466; mean |SHAP| = 0.00342, 0.00339 and 0.00336), and the highest-ranked ProtT5-XL dimension (local index 767, global 1791; mean |SHAP| = 0.00329) ranks fourth. At block level (Figure 3b), the mean |SHAP| per dimension is 7.23 × 10^−4^ for ProtT5-BFD and 6.79 × 10^−4^ for ProtT5-XL, so the two embeddings account for 51.5% and 48.5% of the total attribution, respectively. Several local indices (488, 491, 619 and 767) appear among the top dimensions of both models; because the two embeddings are learned independently, this coincidence is not interpreted further.

These results show that the classifier draws on both ProtT5 embeddings to a nearly equal extent, which is consistent with the higher performance of GAC-BiT-CNN under F-fused features (Acc = 98.34%, AUC = 99.78%, MCC = 0.967) than under either embedding alone. Attribution is broadly distributed: the top 20 dimensions have 3.2–4.9 times the average mean |SHAP| of a dimension (7.01 × 10^−4^) but together account for only 3.7% of the total attribution over the 2048 dimensions, so the prediction depends on many dimensions rather than on a small subset. Because these dimensions are abstract, mean-pooled features, their relationship to specific structural or evolutionary properties of methyltransferases cannot be inferred from SHAP values alone; they do not by themselves identify specific residues, motifs or domains, and no direct biological meaning is assigned to individual dimensions.

### 2.3. Integration of the Model Components

GAC-BiT-CNN is designed as a single integrated network. The multi-scale residual CNN branch learns local patterns from the fused embedding and provides the shared representation used by the other two branches; the bidirectional Transformer branch models long-range dependencies across this representation in both directions; and the capsule (GAC) branch groups the same representation into higher-order capsules through dynamic routing. The three outputs are concatenated and classified jointly, and all branches are trained end-to-end. Because the BiT and GAC branches operate on the output of the CNN branch, removing a branch yields a different, re-trained network rather than isolating that branch’s contribution; a component-wise ablation table is therefore not reported. The benefit of the integrated design is shown by its consistent advantage over recurrent and classical baselines under identical features and evaluation protocol.

### 2.4. Performance Comparison with Machine Learning Classifiers

Table 2 presents the performance of three classical ML classifiers, including AdaBoost, SVM, and LightGBM, evaluated on the same feature space. Even the best-performing ML model, SVM with ProtGen, reached only 85.98% accuracy and an AUC of 94.74%; when evaluated on the fused feature set, SVM accuracy remained approximately 9.5 percentage points below the best GAC-BiT-CNN result. This performance gap highlights the inability of kernel-based and tree-based methods to fully exploit the rich, multi-scale geometric structure encoded in protein language model embeddings, a capacity that the hybrid deep learning architecture of GAC-BiT-CNN is specifically designed to harness.

### 2.5. Performance on the Test/Validation Dataset

GAC-BiT-CNN’s generalisation capability is confirmed by its performance on the held-out test/validation dataset (Table 3). With the fused feature set, GAC-BiT-CNN achieved 95.50% accuracy, 95.23% sensitivity, 95.76% specificity, 95.76% precision, 95.49% F1-score, 98.70% AUC, and 0.901 MCC, outperforming all baselines across every metric.

The strongest competing baseline was RNN with ProtT5-BFD, achieving 91.34% accuracy and 0.827 MCC, leaving a 4.16% accuracy gap. Among recurrent models, RNN consistently outperformed BiLSTM and GRU across most feature descriptors, while BiLSTM and GRU showed degraded performance, particularly on fused features (77.97% and 82.69% accuracy, respectively), suggesting they struggle to integrate heterogeneous feature representations effectively.

The moderate decline from training (98.34%) to test (95.50%) accuracy reflects an expected generalisation gap rather than overfitting. On the independent dataset (93.00% accuracy), specificity is optimistic because its negatives overlap the benchmark (Section 4.1.5). GAC-BiT-CNN maintains superior performance across all feature descriptors on the validation set, with accuracies ranging from 90.88% (ProtGen) to 95.50% (F-fused), demonstrating consistent robustness regardless of feature representation.

#### Performance on the Independent Dataset

To further probe real-world generalisation, GAC-BiT-CNN with the fused feature set was additionally evaluated on the independent dataset of the PMTPred benchmark [13] (Section 4.1.5), which was not used for training, validation or hyperparameter tuning; because its negatives are identical to benchmark sequences, its specificity is optimistic for all models. As shown in Table 4, GAC-BiT-CNN achieved 93.00% accuracy, 89.41% sensitivity, 96.60% specificity, 96.43% precision, 92.78% F1-score, 97.51% AUC, and 0.862 MCC, outperforming all baseline classifiers across every metric.

The best competing baseline, RNN, reached 89.12% accuracy and 0.781 MCC, leaving a 3.88% accuracy gap. BiLSTM (87.11% accuracy) and GRU (86.16% accuracy) performed substantially worse, confirming that GAC-BiT-CNN’s hybrid convolutional-attention architecture with feedback adversarial learning extracts more robust biological patterns than conventional recurrent architectures.

The progressive accuracy decline from training (98.34%) to test (95.50%) to independent (93.00%) evaluation reflects an expected generalisation gap. GAC-BiT-CNN maintains strong discriminative power on the curated Swiss-Prot positives of this set, with 96.60% specificity and 97.51% AUC, confirming exceptional ability to minimise false positives while maintaining high true positive detection, critical for therapeutic target discovery applications. Confusion matrices for the test and independent sets are given in Supplementary Table S4.

### 2.6. Radar Graph Analysis of Models on the Testing Dataset

**Figure 4:**
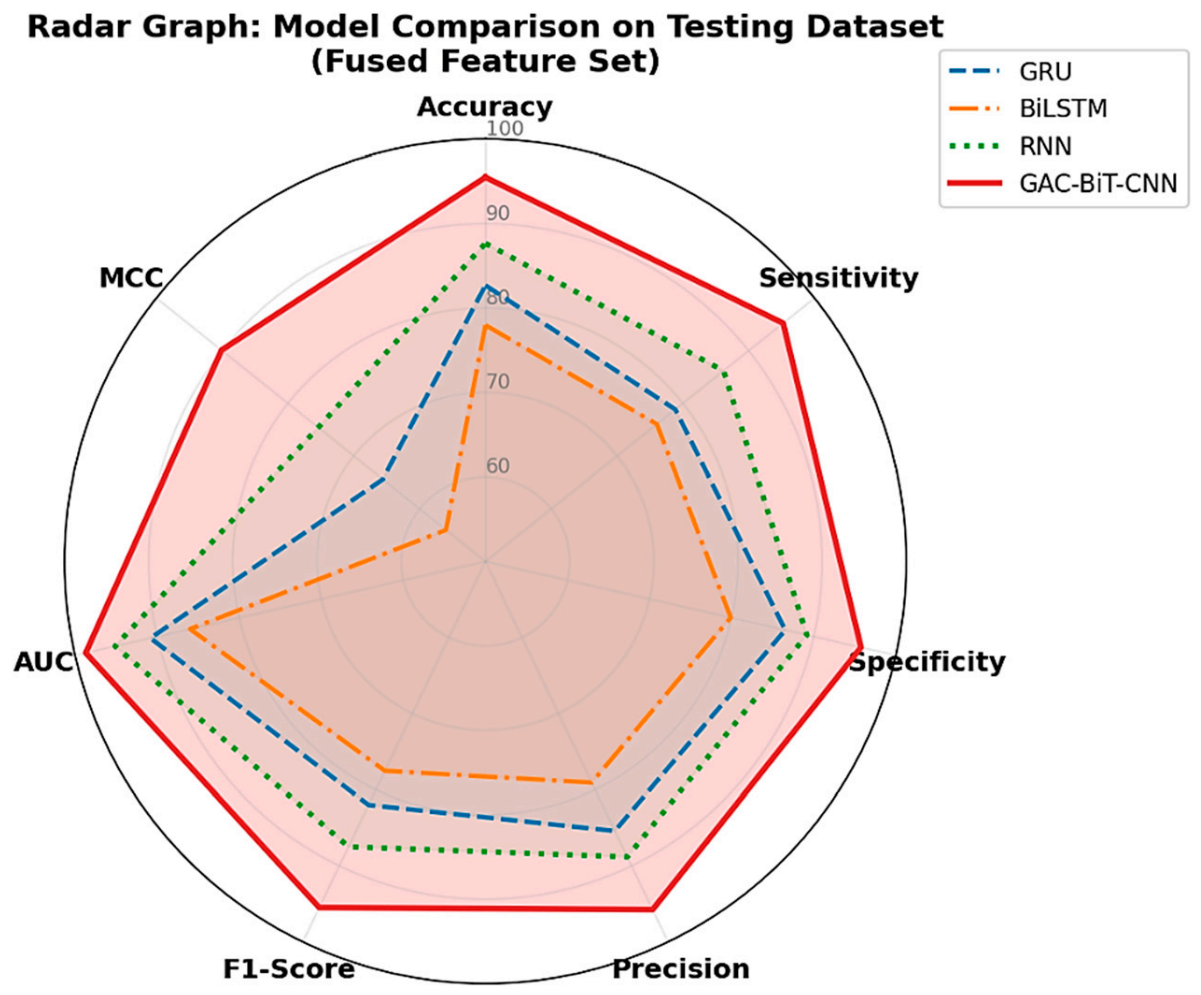
Radar graph comparison of GAC-BiT-CNN against GRU, BiLSTM, and RNN on the test dataset using the fused feature set. Each axis represents one of seven evaluation metrics (normalised to 0–100%). The GAC-BiT-CNN polygon dominates all axes simultaneously.

To provide an intuitive multi-dimensional comparison of model performance on the test dataset, a radar chart was constructed plotting seven evaluation metrics simultaneously: Accuracy, Sensitivity, Specificity, Precision, F1-score, AUC, and MCC. Figure 4 displays the radar profiles for GRU, BiLSTM, RNN, and GAC-BiT-CNN using the fused feature set. The GAC-BiT-CNN polygon extends to the outermost region across all seven axes, demonstrating its uniformly superior and well-balanced performance relative to all competing architectures.

### 2.7. Statistical Significance and Performance Stability

To assess whether the accuracy differences between GAC-BiT-CNN and the competing models are statistically significant, we used a two-sided two-proportion z-test on accuracy (Table 5). McNemar’s test [24], which compares the paired predictions of two classifiers on the same sequences, tests differences in error rate rather than in MCC and requires per-sequence predictions, which were not retained for all baseline models. The two-proportion z-test ignores pairing and is therefore conservative [25]. All comparisons use the same sequences: the 4580 cross-validation predictions for the baselines and the identical 1144-sequence benchmark test set for PMTPred. Differences in MCC are reported only as descriptive effect sizes.

Performance stability was assessed by the standard deviation over the five cross-validation folds and over the five-fold models evaluated on the test set (Table 6). Because only five values are available, 95% confidence intervals were computed with Student’s t-distribution, as mean ± t_0.975,4_ × SD/√5 with t_0.975,4_ = 2.776 [26]. GAC-BiT-CNN showed low variability across all metrics (e.g., cross-validation accuracy 98.34 ± 0.31%).

## 3. Discussion

### 3.1. Comparison with Existing Methods

The performance comparison of the proposed model against the previously published predictor for methyltransferase classification reveals a clear progression in predictive capability, with the proposed GAC-BiT-CNN model demonstrating superior performance, as shown in Table 7.

On the training dataset, PMTPred [13] reports a cross-validation accuracy of 87.02%; sensitivity, specificity, AUC, and MCC were not reported by the original study for this split. The proposed GAC-BiT-CNN model achieves a remarkable 98.34% Acc, 98.39% Sn, 98.29% Sp, 99.78% AUC, and 0.967 MCC, surpassing PMTPred by more than 11 percentage points in accuracy and establishing a strong upper bound on the model’s capacity to learn discriminative sequence patterns.

Similarly, evaluation of the model on the validation dataset is crucial for assessing generalisation. PMTPred [13]—based on an SVM trained on CKSAAP features—shows Acc of 87.94%, Sn of 89.38%, Sp of 88.48%, AUC of 90.40%, and MCC of 0.759. Critically, the proposed GAC-BiT-CNN model maintains a strong lead, achieving 95.50% Acc, 95.23% Sn, 95.76% Sp, 98.70% AUC, and 0.901 MCC on the testing dataset. The consistently higher metrics for GAC-BiT-CNN on the testing set highlight its superior generalisation ability and robustness in identifying methyltransferases compared to the existing method. Because this study uses the PMTPred benchmark without modification, these values refer to the same 1144 test sequences; the published PMTPred sensitivity and specificity imply an accuracy of 88.93% rather than the reported 87.94%, and GAC-BiT-CNN remains 6.57 percentage points more accurate even against the higher value.

Beyond the standard training and validation datasets, robustness on completely unseen sequences was further assessed using a blind independent evaluation. PMTPred [13] reports an accuracy of 86.50%, sensitivity of 82.33%, specificity of 90.67%, AUC of 93.90%, and MCC of 0.733 on its blind dataset of 300 PMT and 300 non-PMT sequences. This is the same independent dataset used in this study, and GAC-BiT-CNN again maintains a clear advantage on it, achieving 93.00% Acc, 89.41% Sn, 96.60% Sp, 97.51% AUC, and 0.862 MCC. This sustained lead under fully blind evaluation conditions confirms that the performance gains of GAC-BiT-CNN are not confined to a single evaluation protocol but generalise consistently across training, testing, and independent assessment regimes. Because the negatives of this set are identical to benchmark sequences, the specificity of both methods is optimistic, and the 7.08-point higher sensitivity on the 300 curated Swiss-Prot positives is the clearest independent evidence of the improvement. PMTPred refers to the benchmark positives as protein methyltransferases, but both studies use the identical benchmark, so the comparison is unaffected by this difference in naming.

Taken together, GAC-BiT-CNN surpasses PMTPred by more than 11 percentage points in training accuracy; on the testing dataset by +7.56% in accuracy, +5.85% in sensitivity, +7.28% in specificity, +8.30% in AUC, and +0.142 in MCC; and on the independent blind dataset by +6.50% in accuracy, +7.08% in sensitivity, +5.93% in specificity, +3.61% in AUC, and +0.129 in MCC. These consistently higher metrics across all three evaluation settings underscore the superior generalisation ability and robustness of GAC-BiT-CNN in predicting methyltransferases, with all improvements found to be statistically significant (Table 5).

### 3.2. Practical Use and Limitations

GAC-BiT-CNN is intended for protein scientists who need to prioritise candidate methyltransferases among newly sequenced or uncharacterised proteins, for example from genome or metagenome projects. Its predictions can be used to triage sequences for biochemical validation and to flag possible annotation errors, and they complement, but do not replace, homology-based annotation (BLAST+ (version 2.12.0), InterPro (version 5.65-97.0)) and experimental assays.

Several limitations should be noted. The task is binary and does not predict the substrate class. The benchmark labels were derived from database annotations, most of which are computationally inferred (only 5.7% of training and test positives are Swiss-Prot reviewed), and some entries are mislabelled, a problem known to affect enzyme superfamilies in public databases [11]. Random label errors act as noise and generally lower measured performance, whereas systematic errors, such as annotations propagated by homology transfer, could lead a model to partly reproduce annotation pipelines; evaluation on the curated independent positives mitigates this concern. In addition, 59.0% of the negatives come from a single genome (*Pseudomonas* sp. AAC) and a composition-only classifier reached 79.46% test accuracy, so part of the separation may reflect organism-specific or compositional signals; the independent set overlaps with the benchmark, so its specificity is optimistic; and SHAP identifies the embedding dimensions the classifier relies on but not the residues or motifs responsible for methyltransferase function. Predictions for individual sequences should therefore be regarded as hypotheses requiring experimental confirmation.

## 4. Material and Methods

### 4.1. Dataset Collection

To ensure a direct comparison with the existing method, this study uses the benchmark datasets of PMTPred (Yadav et al. [13]) as released by the original authors, including its training set (4580 sequences), test set (1144 sequences) and 300/300 independent set; no sequence was added, removed or re-split. Their construction, summarised in Section 4.1.1, Section 4.1.2, Section 4.1.3, Section 4.1.4 and Section 4.1.5, was performed by Yadav et al. [13]; the redundancy and overlap checks reported in Section 4.1.4 and Section 4.1.5 are our own.

#### 4.1.1. Positive Dataset

Positive methyltransferase sequences were retrieved from NCBI using the keyword “Methyltransferase”, yielding 41,146 raw sequences across diverse organisms. Quality filtering was applied to exclude sequences shorter than 50 amino acids and entries annotated as hypothetical, partial, putative, or predicted, resulting in 17,280 annotation-filtered methyltransferase sequences for further processing. A sequence was therefore labelled positive if its NCBI name contained “methyltransferase”, it was not annotated as hypothetical, partial, putative or predicted, it was at least 50 residues long with only standard amino acids, and it was retained by CD-HIT (Section 4.1.3); no Swiss-Prot or Enzyme Commission (EC) requirement was applied. Our check of the released files shows that only 162 of the 2862 training and test positives (5.7%) are reviewed Swiss-Prot entries [27], and that eight positives carry weak annotations (e.g., “possible methyltransferases”). Because of the generic keyword, the positive set contains methyltransferases acting on all classes of substrates (approximately 43.7% generic, 19.8% RNA, 16.0% small-molecule, 13.8% protein and 6.7% DNA methyltransferases; Supplementary Table S3).

#### 4.1.2. Negative Dataset

For the negative class, 33,061 non-methyltransferase sequences were retrieved from NCBI using analogous search criteria. Proteins were sampled across diverse functional categories to avoid functional bias and to ensure that the negative set was broadly representative of general protein sequence space. Sequences containing any keyword associated with methyltransferase activity were strictly excluded from the query. Because the categories and sampling of the original benchmark [13] were not reported in more detail, we characterised the released negative set directly (Supplementary Table S2). The negatives span diverse functions, led by transferases, synthases and ligases other than methyltransferases (599), oxidoreductases and dehydrogenases (243), transcriptional regulators (238), transporters (222) and hydrolases (180). Thirty-three negatives (18 training, 15 test) are nevertheless annotated as methyltransferases; these label errors are inherited from the benchmark and, because they count as false positives when predicted as methyltransferases, the reported specificity is, if anything, slightly underestimated. In addition, 1689 of the 2862 negatives (59.0%) come from a single genome, *Pseudomonas* sp. AAC, which contributes no positives, and only 64 organisms appear in both classes. A logistic regression trained on amino acid composition and sequence length alone reached 79.46% test accuracy (MCC 0.605), compared with 95.50% (MCC 0.901) for GAC-BiT-CNN; these limitations are discussed in Section 3.2.

#### 4.1.3. Preprocessing and Redundancy Reduction

In the benchmark [13], sequence redundancy was reduced using CD-HIT (Cluster Database at High Identity with Tolerance), a program that clusters sequences sharing more than a chosen level of identity and keeps one representative of each cluster, at a 40% sequence identity threshold [28,29,30], applied separately to the complete positive and negative sets before any partitioning. This yielded 2862 methyltransferase and 3009 non-methyltransferase sequences. Sequences shorter than 50 amino acids were discarded as insufficiently informative, and entries containing ambiguous amino acid characters were removed to ensure data consistency and quality. CD-HIT was used solely for redundancy reduction during benchmark dataset construction and is not part of the prediction framework. At inference time, GAC-BiT-CNN operates directly on the raw amino acid sequence without any alignment or homology search, and is therefore alignment-free.

#### 4.1.4. Dataset Partitioning

To construct a balanced binary classification dataset, 2862 non-MT sequences were randomly selected to match the positive class, yielding a final balanced dataset of 5724 sequences. This was randomly partitioned into a training set (4580 sequences: 2290 positive and 2290 negative) and a held-out independent test set (1144 sequences: 572 positive and 572 negative). Because redundancy reduction preceded partitioning, no training–test pair of the same class should share ≥40% identity. We verified this with CD-HIT-2D (version 4.8.1; -c 0.4, -n 2) and BLASTp (2.17.0) [31]: only 4 of the 1144 test sequences (0.35%) share ≥40% identity with a training sequence, and no exact duplicates exist (Supplementary Table S1). All hyperparameter tuning was performed within the training folds, and the test set was used once for final evaluation [32].

#### 4.1.5. Independent Dataset

The independent dataset is the blind set released with the PMTPred benchmark [13]. It contains 600 sequences: 300 methyltransferases selected by Yadav et al. from UniProtKB/Swiss-Prot [27] as well-annotated entries with known three-dimensional structures, and 300 non-methyltransferases. The original authors screened it against the main dataset with CD-HIT-2D, and it was never used for training, model selection or hyperparameter tuning in this study. Our own CD-HIT-2D and BLASTp check showed that the 300 negatives are identical to benchmark sequences (241 training, 59 test) and that 87 positives share ≥40% identity with training sequences (Supplementary Table S1); independent-set specificity should therefore be regarded as optimistic for all methods evaluated on this set.

### 4.2. Feature Extraction via Protein Language Models

Large-scale protein language models learn contextual, evolutionary, and structural representations directly from raw amino acid sequences, requiring neither multiple sequence alignments nor manually engineered features [33,34,35]. Pre-trained on massive sequence databases, these models generate dense, high-dimensional per-sequence embedding vectors that capture long-range residue relationships difficult to encode with conventional feature engineering. In this study, we employ ProtGen, ESM-2, ProtT5-BFD, and ProtT5-XL. Each model is described below.

#### 4.2.1. ProtGen

ProtGen is a generative protein language model based on an encoder–decoder transformer architecture, pre-trained in a self-supervised manner on over 280 million protein sequences from the UniRef90 and Big Fantastic Database (BFD) databases [36]. Unlike discriminative models trained on labelled data, ProtGen learns the joint probability distribution over amino acid positions, enabling the generation of biologically plausible protein sequences. For a sequence *S* = (*a*_1_, *a*_2_, …, *a_L_*), the generative probability is factorised as:

(1) $$P(S)=\prod_{i}^{n}P\left(a_{i}|a_{1},\ldots ,a_{i-1}\right)$$

in which all the conditional probabilities are parameterised by transformer attention layers. The overall per-sequence representation is the mean of encoder hidden states:

(2) $$e_{\mathrm{ProtGen}}=\frac{1}{L}\sum_{i}^{n}h_{i}^{\mathrm{encoder}}\in R^{1536}$$

Advantage: ProtGen generalises effectively to evolutionarily distant sequences, capturing broad protein sequence diversity. Its alignment-free generative pre-training enables the exploration of novel sequences without requiring curated homologue data, making it particularly valuable for identifying methyltransferase families with atypical sequence compositions across taxonomic groups.

#### 4.2.2. ESM-2

ESM-2 is developed by Meta AI Research, trained on over 250 million unique protein sequences from UniRef90 using a masked language modelling (MLM) objective [17]. Given a sequence S and masked elements M ⊆ {1, …, L} the pre-training loss is:

(3) $$L_{\mathrm{MLM}}=-\sum_{i\in M}^{n}\mathrm{log P}\left(a_{i}|S_{\mathrm{masked}}\right)$$

The self-attention mechanism of N transformer layers produces contextualised per-residue embeddings, h_i_ ∈ ℝ, where d = 1280 for the ESM-2-650M variant of the embeddings employed in the study. A fixed-length sequence representation is obtained via mean pooling:

(4) $$e_{\mathrm{ESM}2}=\frac{1}{L}\sum_{i}^{n}h_{i}\in R^{1280}$$

Advantage: Trained on 250 million protein sequences, ESM-2 implicitly learns evolutionary constraints and structural residue–residue contacts, producing state-of-the-art sequence representations. In our experiments, ESM-2 consistently yielded the highest performance among individual PLM feature sets for GAC-BiT-CNN.

#### 4.2.3. ProtT5-BFD

ProtT5-BFD, a Text-to-Text Transfer Transformer (T5)-based model, is trained on the Big Fantastic Database (BFD), encompassing over 2.1 billion protein sequences from metagenomic samples and complete proteomes [18]. In the case of masked positions M, the denoising loss is:

(5) $$L_{\mathrm{denoise}}=-\sum_{i\in M}^{n}\mathrm{log P}\left(a_{i}|S_{\mathrm{masked}}\right)$$

The T5 encoder represents the input sequence S in contextualised embeddings with two layers of bidirectional attention, producing per-sequence embeddings of dimension d = 1024:

(6) $$e_{\mathrm{BFD}}=\frac{1}{L}\sum_{i}^{n}h_{i}^{T5}\in R^{1024}$$

Advantage: By virtue of training on a 2.1 billion sequence corpus that includes metagenomic and environmental sequences absent from curated databases such as UniRef, ProtT5-BFD provides exceptional taxonomic breadth and enables robust representation of novel or poorly characterised methyltransferase subfamilies.

#### 4.2.4. ProtT5-XL

ProtT5-XL is a variant of the ProtT5 family trained on UniRef50, a non-redundant subset of UniProt clustered at 50% sequence identity [18]. The T5 encoder encodes the sequence in a stack of bidirectional self-attention layers:

(7) $$\mathrm{Attention}\left(Q,K,V\right)=\mathrm{softmax}\left(\frac{QK^{T}}{\sqrt{d_{k}}}\right)V$$

where Q, K, and V are query, key, and value matrices derived from input embeddings, and d_k_ is the key vector dimension. For the XL variant, d = 1024, yielding:

(8) $$e_{\mathrm{XL}}=\frac{1}{L}\sum_{i}^{n}h_{i}^{\mathrm{xl}}\in R^{1024}$$

Advantage: Training on the high-quality, non-redundant UniRef50 database enables ProtT5-XL to achieve superior precision on well-characterised methyltransferase families, and to resolve structurally conserved motifs found in SAM-binding proteins, including Rossmann folds.

#### 4.2.5. Fused Feature Set and Dimensionality

A fused feature set was constructed by concatenating the per-sequence embedding vectors of ProtT5-BFD and ProtT5-XL (2 × 1024 = 2048 dimensions); the details are provided in Table 8.

*F*_fused_ = [*e*_BFD_ ‖ *e*_XL_] ∈ ℝ^2048^ (9)

### 4.3. Proposed Model: GAC-BiT-CNN

GAC-BiT-CNN is a hybrid DL architecture designed for accurate, alignment-free methyltransferase identification from PLM embeddings. The model processes input protein embeddings through three branches, i.e., CNN, BiT, and GAC; the BiT and GAC branches operate on the feature map produced by the CNN branch, and each branch produces a distinct representation. The resulting branch outputs are concatenated and passed through fully connected layers to yield a binary classification decision (MT vs. non-MT).

#### 4.3.1. Generative Adversarial Capsule Module

To enhance generalisation and robustness to uncharacterised methyltransferase sequences, the GAC branch incorporates a generative adversarial network (GAN) operating at the protein embedding level. The GAN is trained adversarially using two sub-networks: a Generator G, which synthesises embedding-space samples from random noise vectors z~p(z), and a Discriminator D, which distinguishes real from synthetic samples. The adversarial minimax objective is:

(10) $$\min_{G}\;\max_{D}\;V\left(D,G\right)=E\left[\log\;D\left(x\right)\right]+E\left[\log\;\left(1-D\left(G\left(z\right)\right)\right)\right]$$

The GAN-refined embeddings are subsequently processed by Capsule Networks, which model hierarchical spatial relationships and inter-feature dependencies. Dynamic routing between primary and digit capsules is performed as follows:

(11) $$v_{j}=\mathrm{squash}\left(\sum_{i}^{n}c_{\mathrm{ij}}\;{û}_{j|i}\right)$$

(12) $$\mathrm{squash}\left(s_{j}\right)=\frac{{\left|s_{j}\right|}^{2}}{1+{\left|s_{j}\right|}^{2}}\cdot \frac{s_{j}}{\left|s_{j}\right|}$$

Here, c_ij_ are dynamically agreed-upon routing coefficients, û_j_|_i_ is the prediction vector of capsule i to capsule j, and squash is the function that buffers the output vectors of the capsules to the probability of the existence of the entity [37,38].

#### 4.3.2. Convolutional Feature Extraction

Each feature stream is passed through a dedicated CNN block that functions as a local feature extractor. The CNN branch applies 1D convolutional filters with multiple kernel sizes (3, 5, 7) and dilation rates (1, 2, 3), with rectified linear unit (ReLU) activation, to capture local patterns at varying receptive field scales. Because the input is a mean-pooled embedding, these convolutions slide over embedding dimensions rather than residue positions; residue-level context and motifs are captured upstream by the self-attention layers of each PLM before pooling, so the filters act as learned local feature-interaction detectors rather than motif detectors:

*x_k_* = ReLU(Conv1D(*F*_fused_; kernel = *k*, dilation = *d*)) (13)

A residual connection is added after every block of convolutions to help gradient propagation in deep networks and avoid vanishing gradients, and to keep the low-level features, as demonstrated in related tasks [39,40]:

*x*_out_ = *x_k_* + *x*_shortcut_ (14)

#### 4.3.3. Bidirectional Transformer Branch

The BiT branch extends the standard transformer architecture to process protein embeddings bidirectionally, simultaneously encoding both forward and backward sequence context. Multi-head self-attention (MHA) [41] is applied to the feature map of the CNN branch, in which each position corresponds to one embedding dimension; it therefore captures dependencies between embedding dimensions, i.e., interactions within and across the input embeddings, rather than residue–residue relationships:

(15) $$\mathrm{Attention}\left(Q,K,V\right)=\mathrm{softmax}\left(\frac{QK^{T}}{\sqrt{d_{k}}}\right)V$$

where ℝ*Q* = *XW_Q_*, *K* = *XW_K_*, *V* = *XW_V_*, and *W_Q_*, *W_K_*, *W_V_* ∈ ℝ*^d^*^×*d*^*_k_* are learned projection matrices. A feed-forward network (FFN) sublayer with residual connection follows each attention block:

FFN(*x*) = ReLU(*xW*_1_ + *b*_1_)*W*_2_ + *b*_2_ (16)

The BiT output combines forward and backward passes, consistent with prior work on bidirectional sequence models [42]:

BiT_output_ = TCN_forward_(*x*) + TCN_backward_(*x*) (17)

#### 4.3.4. Feature Fusion and Classification

The outputs of the CNN, BiT, and GAC branches are concatenated to form a unified feature vector:

*F*_concat_ = [*F*_CNN_ ‖ *F*_BiT_ ‖ *F*_GAC_] (18)

A sigmoid activation function in the output layer converts the fused representation into a probability score for binary classification:

*Y* = sigmoid(*W* · *F*_concat_ + *b*) (19)

where W and b are learned weights and biases. A threshold of 0.5 converts the probability score into binary predictions (MT vs. non-MT).

### 4.4. Hyperparameter Tuning of the GAC-BiT-CNN

All components of the GAC-BiT-CNN architecture were subjected to systematic hyperparameter optimisation via grid search. To prevent data leakage, all hyperparameter searches were conducted exclusively within the training data of each cross-validation fold. Table 9 summarises the hyperparameter search space and best-found values for the components of GAC-BiT-CNN.

### 4.5. Machine Learning Hyperparameter Tuning

Classical machine learning (ML) classifiers (Adaptive Boosting (AdaBoost), SVM and Light Gradient Boosting Machine (LightGBM)) were also implemented to evaluate the performance and compare with the proposed model. In this connection, a similar grid search is applied to select the best values for hyperparameters. Table 10 presents the search ranges and best values identified for each classifier.

### 4.6. Model Validation and Performance Metrics

Five-fold cross-validation, a resampling technique in which the dataset is randomly partitioned into five equal and mutually exclusive subsets (folds), was employed to rigorously evaluate model performance and reduce the risk of overfitting. In each of the five iterations, four folds are used for model optimisation while the remaining fold is held out for validation, cycling through all possible combinations until every fold has served as the validation set exactly once. By averaging performance metrics across all five folds, the approach reduces variance in the final evaluation and offers a more reliable assessment than a single train-validation split [43]. It was evaluated with seven evaluation metrics from the binary confusion matrix:

(20) $$\mathrm{Acc}=\frac{\mathrm{TP}+\mathrm{TN}}{\mathrm{TP}+\mathrm{FN}+\mathrm{TN}+\mathrm{FP}}$$

(21) $$\mathrm{Sn}=\frac{\mathrm{TP}}{\mathrm{TP}+\mathrm{FN}}$$

(22) $$\mathrm{Sp}=\frac{\mathrm{TN}}{\mathrm{TN}+\mathrm{FP}}$$

(23) $$\mathrm{MCC}=\frac{\mathrm{TP}\times \mathrm{TN}-\mathrm{FP}\times \mathrm{FN}}{\sqrt{\left(\mathrm{TP}+\mathrm{FP})(\mathrm{TN}+\mathrm{FP})(\mathrm{TN}+\mathrm{FN})(\mathrm{TP}+\mathrm{FN}\right)}}$$

(24) $$\mathrm{Pre}=\frac{\mathrm{TP}}{\mathrm{TP}+\mathrm{FP}}$$

(25) $$F1-\mathrm{score}=\frac{2\times \mathrm{Pre}\times \mathrm{Recall}}{\mathrm{Pre}+\mathrm{Recall}}$$

(26) $$\mathrm{AUC}=\int_{0}^{1}\mathrm{TPR}\left(\mathrm{FPR}\right)d\left(\mathrm{FPR}\right)$$

Among these, MCC is considered the most informative metric for binary classification, as it is robust to class imbalance and incorporates all four elements of the confusion matrix [44]. An AUC of 1.0 indicates perfect class separation, whereas an AUC of 0.5 is equivalent to random classification.

## 5. Conclusions

This study presents GAC-BiT-CNN, a novel hybrid deep learning model for alignment-free, high-throughput methyltransferase prediction from protein language model embeddings. With the fused feature set, GAC-BiT-CNN achieved 98.34% accuracy (AUC 99.78%) in five-fold cross-validation on the training set, 95.50% accuracy (AUC 98.70%, MCC 0.901) on the held-out test set, and 93.00% accuracy (AUC 97.51%, MCC 0.862) on the independent dataset of Yadav et al., whose specificity is optimistic because of its overlap with the benchmark. On the identical benchmark test set, it exceeded PMTPred by 7.56 percentage points in accuracy (95.50% vs. 87.94%). SHAP analysis provides representation-level attribution of the fused embedding; it does not by itself identify biologically meaningful residues or motifs. Radar chart analysis confirms the consistent advantage of the integrated architecture over the recurrent baselines. Future work will explore: (i) multi-task learning for subtype-level classification of methyltransferases (PRMT vs. PKMT vs. DNMT); (ii) integration of AlphaFold2 [45] structural embeddings to augment sequence-level MT representations; (iii) application of contrastive learning and multi-view fusion to further strengthen discriminative representations; (iv) development of a publicly accessible web server for community use; and (v) benchmarking on newly deposited large-scale methyltransferase datasets to assess generalisation beyond the current evaluation set. In addition, applying the convolutional and attention branches to per-residue embeddings instead of pooled vectors would allow genuine residue-level motif learning and attribution.

## Figures and Tables

**Table 1:** Performance of DL classifiers on the training dataset.

Classifier	Feature	Acc (%)	Sn (%)	Sp (%)	Pre (%)	F1 (%)	AUC (%)	MCC
GRU	ProtGen	88.14	89.00	87.31	87.59	88.25	94.24	0.764
	ESM-2	82.28	84.69	81.05	81.72	83.16	90.91	0.658
	ProtT5-BFD	88.93	89.95	87.92	88.15	89.04	95.72	0.779
	ProtT5-XL	88.93	89.96	87.91	88.15	89.04	95.72	0.779
	F-fused	87.00	88.43	85.59	86.01	87.18	93.95	0.741
BiLSTM	ProtGen	89.83	89.91	89.75	89.73	89.82	95.74	0.797
	ESM-2	82.21	83.03	81.34	81.65	82.31	90.60	0.644
	ProtT5-BFD	85.20	84.87	85.52	85.44	85.14	92.74	0.704
	ProtT5-XL	85.26	85.56	84.99	85.07	85.29	92.50	0.706
	F-fused	83.73	84.70	82.76	83.08	83.88	91.42	0.675
RNN	ProtGen	92.21	92.55	91.89	91.93	92.23	97.70	0.844
	ESM-2	85.93	86.19	85.65	85.74	85.96	93.16	0.718
	ProtT5-BFD	92.21	92.55	91.89	91.93	92.23	97.70	0.844
	ProtT5-XL	88.10	88.77	87.41	87.65	88.19	94.58	0.762
	F-fused	88.95	90.32	87.57	87.96	89.12	95.19	0.779
GAC-BiT-CNN	ProtGen	96.42	95.96	96.85	96.84	96.39	99.40	0.929
	ESM-2	97.84	97.60	98.08	98.07	97.83	99.76	0.957
	ProtT5-BFD	97.99	97.60	98.39	98.37	97.98	99.71	0.960
	ProtT5-XL	98.03	97.99	98.07	98.09	98.04	99.72	0.961
	F-fused	98.34	98.39	98.29	98.31	98.34	99.78	0.967

**Table 2:** Performance of classical ML classifiers.

Classifier	Acc (%)	Sn (%)	Sp (%)	Pre (%)	F1 (%)	AUC (%)	MCC
AdaBoost	85.46	88.49	82.39	83.43	85.86	93.47	0.710
SVM	85.98	82.59	89.40	88.65	85.48	94.74	0.720
LightGBM	82.12	90.05	74.26	77.95	83.47	91.48	0.651
GAC-BiT-CNN	98.34	98.39	98.29	98.31	98.34	99.78	0.967

**Table 3:** Performance of DL classifiers on the validation dataset. For GAC-BiT-CNN, accuracy, precision, F1-score and MCC were recomputed from sensitivity and specificity; the F-fused row is the mean over the five-fold models.

Classifier	Feature Descriptor	Acc (%)	Sn (%)	Sp (%)	Pre (%)	F1-Score (%)	AUC (%)	MCC
GRU	ProtGen	84.96	85.53	84.61	84.72	85.01	92.65	0.699
	ESM-2	81.30	87.93	78.67	80.48	84.04	91.85	0.669
	ProtT5-BFD	86.80	85.66	87.93	87.65	86.64	93.13	0.736
	ProtT5-XL	84.44	82.51	86.36	85.81	84.13	92.58	0.689
	F-fused	82.69	78.84	86.53	85.41	82.00	90.82	0.656
BiLSTM	ProtGen	86.80	86.01	87.58	87.38	86.69	93.78	0.736
	ESM-2	84.00	88.63	79.37	81.12	84.71	93.19	0.683
	ProtT5-BFD	85.75	84.96	86.53	86.32	85.63	93.56	0.715
	ProtT5-XL	77.62	72.90	82.34	80.50	76.51	86.31	0.555
	F-fused	77.97	76.04	79.89	79.09	77.54	85.99	0.560
RNN	ProtGen	86.53	83.91	89.16	88.56	86.17	93.78	0.732
	ESM-2	85.92	86.18	85.66	85.73	85.96	93.16	0.719
	ProtT5-BFD	91.34	89.68	93.00	92.76	91.20	96.92	0.827
	ProtT5-XL	80.50	78.84	82.16	81.55	80.17	88.60	0.610
	F-fused	87.67	86.18	89.16	88.82	87.48	95.13	0.754
GAC-BiT-CNN	ProtGen	90.88	86.73	95.02	94.57	90.48	97.74	0.820
	ESM-2	94.73	94.72	94.73	94.73	94.72	97.03	0.895
	ProtT5-BFD	93.29	93.10	93.49	93.46	93.28	98.60	0.866
	ProtT5-XL	95.08	94.90	95.26	95.24	95.07	98.50	0.902
	F-fused	95.50	95.23	95.76	95.76	95.49	98.70	0.901

**Table 4:** Performance of GAC-BiT-CNN on the independent dataset. MCC values of GRU, BiLSTM and RNN were recomputed from sensitivity and specificity.

Classifier	Acc (%)	Sn (%)	Sp (%)	Pre (%)	F1-Score (%)	AUC (%)	MCC
GRU	86.16	83.08	87.33	85.87	84.73	89.82	0.705
BiLSTM	87.11	84.36	89.61	87.32	86.11	91.69	0.741
RNN	89.12	85.54	92.35	91.96	88.23	93.77	0.781
GAC-BiT-CNN	93.00	89.41	96.60	96.43	92.78	97.51	0.862

**Table 5:** Significance of accuracy differences between GAC-BiT-CNN and competing methods (two-sided two-proportion z-test; ΔMCC is descriptive and was not tested).

Comparison	Validation Method	Acc GAC-BiT-CNN (%)	Acc Competitor (%)	ΔAcc	z	*p*-Value	ΔMCC
vs. GRU	5-fold CV (4580)	98.34	87.00	+11.34	20.82	<10^−95^	+0.226
vs. BiLSTM	5-fold CV (4580)	98.34	83.73	+14.61	24.47	<10^−131^	+0.292
vs. RNN	5-fold CV (4580)	98.34	88.95	+9.39	18.42	<10^−75^	+0.188
vs. SVM	5-fold CV (4580)	98.34	85.98	+12.36	22.00	<10^−106^	+0.247
vs. PMTPred	Test set (1144)	95.50	87.94	+7.56	6.56	5.3 × 10^−11^	+0.142

**Table 6:** Performance stability and 95% confidence intervals for GAC-BiT-CNN across 5-fold cross-validation and the held-out test dataset. Confidence intervals use Student’s t-distribution (df = 4).

Metric	Dataset	Mean ± Std	95% Confidence Interval
Accuracy (%)	5-Fold CV	98.34 ± 0.31	[97.96, 98.72]
	Test Dataset	95.50 ± 0.44	[94.95, 96.05]
Sensitivity (%)	5-Fold CV	98.39 ± 0.35	[97.96, 98.82]
	Test Dataset	95.23 ± 0.51	[94.60, 95.86]
Specificity (%)	5-Fold CV	98.29 ± 0.38	[97.82, 98.76]
	Test Dataset	95.76 ± 0.39	[95.28, 96.24]
MCC	5-Fold CV	0.967 ± 0.006	[0.960, 0.974]
	Test Dataset	0.901 ± 0.009	[0.890, 0.912]

**Table 7:** Comparison of GAC-BiT-CNN with existing methods (n.r. = not reported in the original study).

Method	Feature Set	Acc (%)	Sn (%)	Sp (%)	AUC (%)	MCC
**Training dataset**						
PMTPred [13]	CKSAAP + SVM	87.02	n.r.	n.r.	n.r.	n.r.
GAC-BiT-CNN (Proposed)	Fused PLM Embeddings	98.34	98.39	98.29	99.78	0.967
**Validation dataset**						
PMTPred [13]	CKSAAP + SVM	87.94	89.38	88.48	90.40	0.759
GAC-BiT-CNN (Proposed)	Fused PLM Embeddings	95.50	95.23	95.76	98.70	0.901
**Independent (Blind) Dataset**						
PMTPred [13]	CKSAAP + SVM	86.50	82.33	90.67	93.90	0.733
GAC-BiT-CNN (Proposed)	Fused PLM Embeddings	93.00	89.41	96.60	97.51	0.862

**Table 8:** Summary of protein language model embedding dimensions and pre-training databases used in this study.

Feature Set	Embedding Dimension	Pre-Training Database
ProtGen	1536	UniRef90 + BFD (280M sequences)
ESM-2	1280	UniRef90 (250M sequences)
ProtT5-BFD	1024	BFD (2.1B sequences; metagenomics)
ProtT5-XL	1024	UniRef50 (curated, non-redundant)
F-fused	2048	ProtT5-BFD and ProtT5-XL concatenated

**Table 9:** Hyperparameter search space and optimal configuration for GAC-BiT-CNN.

Component	Hyperparameter	Search Range	Best Value
Capsule Network	Primary Capsule Dim (d_p_)	[4, 8, 16, 32]	8
	Num. Capsules	[2, 4, 8]	4
	Routing Iterations (r)	[2, 3, 5]	3
	Capsule Dropout	[0.1, 0.2, 0.3]	0.2
CNN Branch	Kernel Sizes	[(3, 5), (3, 5, 7), (3, 7, 11)]	(3, 5, 7)
	Dilation Rates	[(1, 2), (1, 2, 3), (1, 2, 4)]	(1, 2, 3)
	Filters per Conv	[32, 64, 128]	64
BiT Branch	Num. Attention Heads	[2, 4, 8]	2
	Key Dimension (d_k_)	[16, 32, 64]	32
	LSTM Hidden Units	[32, 64, 128]	64
GAN	Generator LR (ηG)	[0.0001, 0.0005, 0.001]	0.0005
	Discriminator LR (ηD)	[0.00005, 0.0001, 0.0005]	0.0001
	Adversarial Loss Weight (λ)	[0.5, 1.0, 2.0]	1.0
Training	Batch Size (B)	[16, 32, 64]	32
	Learning Rate	[1 × 10^−5^, 1 × 10^−4^, 5 × 10^−4^]	1 × 10^−4^
	Epochs (max)	[50, 100, 200]	100 (early stop)
	Dense Layer Units	[64, 128, 256]	128
	Dropout Rate	[0.2, 0.3, 0.5]	0.3

**Table 10:** Hyperparameter search ranges and best values for classical machine learning baselines.

Model	Hyperparameter	Search Range	Best Value
SVM	C (Regularisation)	[0.1, 1, 10, 100]	10
	Gamma (kernel coeff.)	[0.001, 0.01, 0.1, 1]	0.01
	Kernel	[‘linear’, ‘rbf’, ‘poly’]	‘rbf’
AdaBoost	n_estimators	[50, 100, 200, 500]	200
	Learning Rate	[0.01, 0.1, 0.5, 1.0]	0.1
	Base Estimator Depth	[1, 2, 3]	2
LightGBM	n_estimators	[100, 200, 500]	200
	Learning Rate	[0.01, 0.05, 0.1]	0.05
	Max Depth	[−1, 6, 10, 15]	6
	num_leaves	[31, 63, 127]	63
	Min child samples	[10, 20, 50]	20

## Data Availability

The datasets and source code supporting the findings of this study are freely available at https://github.com/MuhammadMuazzam/GAT-BiT-CNN (accessed on 4 October 2026).

## Supplementary Materials

The following supporting information can be downloaded at: https://www.mdpi.com/article/10.3390/ijms27219967/s1.

## Abbreviations

The following abbreviations are used in this manuscript:

AdaBoost	Adaptive Boosting
AUC	Area under the receiver operating characteristic curve
BFD	Big Fantastic Database
BiLSTM	Bidirectional long short-term memory
BiT	Bidirectional Transformer
CD-HIT	Cluster Database at High Identity with Tolerance
CKSAAP	Composition of k-spaced amino acid pairs
CNN	Convolutional neural network
DL	Deep learning
EC	Enzyme Commission
ESM	Evolutionary Scale Modeling
FFN	Feed-forward network
FPR	False positive rate
GAC	Generative adversarial capsule
GAN	Generative adversarial network
GRU	Gated recurrent unit
HMM	Hidden Markov model
LightGBM	Light Gradient Boosting Machine
MCC	Matthews correlation coefficient
MHA	Multi-head self-attention
ML	Machine learning
MT	Methyltransferase
NCBI	National Centre for Biotechnology Information
PKMT	Protein lysine methyltransferase
PLM	Protein language model
PMT	Protein methyltransferase
PRMT	Protein arginine methyltransferase
ReLU	Rectified linear unit
RNN	Recurrent neural network
RNMT	RNA methyltransferase
ROC	Receiver operating characteristic
SAM	S-adenosyl-L-methionine
SHAP	SHapley Additive exPlanations
SVM	Support vector machine
T5	Text-to-Text Transfer Transformer
TCN	Temporal convolutional network
TPR	True positive rate
UniProtKB	UniProt Knowledgebase
