# Supplementary material for: GAC-BiT-CNN: A Hybrid Generative Adversarial Capsule–Transformer–CNN Model with Fused Protein Language Model Embeddings for Methyltransferase Prediction
Source: Int J Mol Sci. 2026 Nov 6;27(21):9967. doi: 10.3390/ijms27219967 (PMC13764292; doi:10.3390/ijms27219967)
Supplement: Supplementary file 1 [file ijms-27-09967-s001.zip › ijms-4640697-supplementary.pdf]

Supplementary Materials

GAC-BiT-CNN: A Hybrid Generative Adversarial Capsule–Transformer–CNN Model with Fused Protein Language Model Embeddings for Methyltransferase Prediction

Muhammad Muazzam [1], Farman Ali [1,*], Raed Alsini [2], Naif Waheb Rajkhan [3], Omar Alghushairy [4] and Tamim Alkhalifah [5,*]

** Correspondence: farman335@yahoo.com or farman.buic@bahria.edu.pk (F.A.); tkhliefh@qu.edu.sa (T.A.)*

Table S1. Sequence overlap between the benchmark datasets (CD-HIT-2D v4.8.1, -c 0.4 -n 2; BLASTp, E-value ≤ 10^{-3}, coverage ≥ 50%).

Comparison	CD-HIT-2D (≥40% identity)	Exact duplicates	BLASTp closest training match
Test (1144) vs. training	4 (0.35%; all negatives)	0	median 35.2%, max 77.7%
Independent positives (300) vs. training	87 (29.0%)	0	133 sequences ≥40%
Independent negatives (300) vs. training + test	300 (100%)	300 (241 training, 59 test)	100% identity

CD-HIT-2D, Cluster Database at High Identity with Tolerance (two-database comparison); BLASTp, Basic Local Alignment Search Tool for proteins (BLAST+ version 2.12.0). The independent dataset is the blind set of the PMTPred benchmark.

Table S2. Functional categories of the 2862 benchmark negatives (assigned from annotation text).

Category	Number
Transferases, synthases and ligases (non-MT)	599
Oxidoreductases / dehydrogenases	243
Transcriptional regulators / DNA-binding	238

Category	Number
Transporters / channels / permeases	222
Hydrolases / peptidases / esterases	180
Kinases / signalling	148
Lyases / isomerases	113
Hypothetical / domain-only	45
Ribosomal / translation	38
Other or mixed annotations	1036
Annotated as methyltransferase (label errors; included above)	33

MT, methyltransferase. Categories were assigned by keyword matching of the annotation text; the 33 negatives annotated as methyltransferases are counted within the categories above.

Table S3. Composition of the 2862 training and test positives by substrate class (assigned from annotation text; counts are approximate).

Substrate class	Examples of annotations	Number	%
Unassigned (generic)	“methyltransferase”, “SAM-dependent methyltransferase”	1250	43.7
RNA MTs	rRNA MT, tRNA (uracil-5-)-MT, METTL	567	19.8
Small-molecule MTs	O-methyltransferase, ubiquinone/menaquinone MT	458	16.0
Protein MTs	histone-lysine N-MT, arginine N-MT, SET-domain	396	13.8

Substrate class	Examples of annotations	Number	%
DNA MTs	DNA (cytosine-5)-MT, adenine-specific MT	191	6.7
Total		2862	100

MT, methyltransferase; METTL, methyltransferase-like; SAM, S-adenosyl-L-methionine; SET, Su(var)3-9, Enhancer-of-zeste and Trithorax domain.

Table S4. Confusion matrices of GAC-BiT-CNN (fused features), mean counts over the five fold models.

Dataset	TP	FN	TN	FP	Acc (%)	Sn (%)	Sp (%)
Test (572 MT / 572 non-MT)	544.7	27.3	547.7	24.3	95.50	95.23	95.76
Independent (300 MT / 300 non-MT)	268.2	31.8	289.8	10.2	93.00	89.41	96.60

TP, true positives; FN, false negatives; TN, true negatives; FP, false positives; Acc, accuracy; Sn, sensitivity; Sp, specificity. Counts are means over the five models trained in five-fold cross-validation, so they are not whole numbers.
